# A Novel Culture System for Inhibiting In Vitro Differentiation of Ovine Granulosa Cells

**DOI:** 10.3390/biom15091280

**Published:** 2025-09-04

**Authors:** Yufen Zhao, Haijiang Liu, Zhe Mu, Haijun Li, Wangmei Qi

**Affiliations:** 1 College of Veterinary Medicine, Inner Mongolia Agricultural University, Hohhot 010018, China; 18686115060@emails.imau.edu.cn (Y.Z.); haijiangliu@emails.imau.edu.cn (H.L.); zhemu@emails.imau.edu.cn (Z.M.); 2 Key Laboratory of Animal Embryo and Development Engineering of Autonomous Region Universities, Inner Mongolia Agricultural University, Hohhot 010018, China; 3 Inner Mongolia Key Laboratory of Veterinary Fundamentals and Disease Control of Herbivorous Livestock, Inner Mongolia Agricultural University, Hohhot 010018, China

**Keywords:** sheep, granulosa cell, luteinization, FSHR, STAR, LHR antagonist

## Abstract

The in vitro granulosa cell (GC) model presents a valuable tool to explore antral follicle development. A full understanding of the reasons and blocking methods that occur during in vitro luteinization of sheep GCs, stimulated by serum culture, is a complex goal that has not been completely achieved. Herein, the phenomenon and causes of GC differentiation, as well as the methods for inhibiting luteinization in an in vitro culture system, were investigated by immunofluorescence, Western blot, RT-qPCR, and ELISA techniques. The results reveal that, when compared to fresh GCs, FSHR protein levels in primary GCs significantly decreased in serum-containing media, while STAR protein levels significantly increased, implying that sheep GCs can differentiate in serum-containing media. LH concentrations were significantly greater in serum-containing media compared to serum-free media. The LH receptor (LHR) mRNA expression in primary-generation GCs steadily increased with longer culture times, indicating that LH-LHR signaling leads to GC luteinization in vitro. In primary and second-generation GCs, 180 nmol/L BAY-899, an LHR-specific antagonist, significantly increased FSHR protein expression, reduced STAR protein synthesis, and inhibited P4 secretion within 48 h of in vitro culture compared to controls. BAY-899 showed no adverse effects on fifth-generation GCs growth, implying that BAY-899 can inhibit GC luteinization while not affecting cell proliferation. In conclusion, this study found that the LHR antagonist BAY-899 can preserve the features of sheep GCs in vitro by suppressing the spontaneous luteinization process caused by LH-LHR signaling, which has a key methodological implication for studying the mechanics of antral follicle formation in vivo.

## 1. Introduction

Mammals experience a distinct developmental stage known as the antral follicle stage, which precedes ovulation. During this stage, granulosa cells (GCs) actively proliferate and exchange nutrients and information with the oocyte via gap junctions, playing a critical role in supporting oocyte maturation [1]. Notably, research indicates that GC activity peaks in small antral follicles [2]. Given these attributes, isolating GCs from early antral follicles and culturing them in an appropriate in vitro environment serves as an excellent model for investigating GC proliferation and apoptosis. This approach offers significant methodological value for exploring the developmental processes of early antral follicles in sheep.

Using an in vitro GCs growth model, studies found that both shorter and longer incubation period enabled the GCs to undergo a spontaneous luteinization under serum conditions, which are associated with functional and morphological differentiation. Pescador et al. demonstrated that P450arom mRNA was undetectable in pig GCs cultured for 24 h in 10% serum, while P450scc mRNA levels significantly increased [3]. After a 48 h culture period with serum, the GCs exhibited morphological characteristics indicative of luteinization, thereby transforming into luteinized GCs with core features: enlarged size, polygonal shape, and increased cytoplasmic lipid droplets [4]. Similarly, M. Breckwoldt and colleagues observed that after 72 h of in vitro culture with 5% serum, human GCs exhibited ultrastructural changes, including numerous lipid droplets and well-developed mitochondria [5]. Studies focusing on sheep GC proliferation, apoptosis, and associated steroid hormones have largely neglected the question of GC luteinization in these conditions [6,7]. Furthermore, previous research has primarily relied on the specific expression of follicle-stimulating hormone receptor (FSHR) protein to identify GCs, often overlooking other indicators of luteinization [8]. Therefore, using luteinized GCs as a model for studying early antral follicle development would be inappropriate. To address this gap, it is crucial to establish a non-luteinized in vitro GC culture model. Such a model is essential for effectively investigating the developmental mechanisms of early antral follicles and ensuring the accuracy of experimental findings in this domain.

Previous studies attempted to culture GCs in serum-free media to create a non-luteinized GCs system for obtaining undifferentiated GCs. Serum is a critical additive in the in vitro culture of mammalian cells, providing essential nutrients and signaling molecules necessary for cell growth [9,10]. However, the GCs derived from serum-free cultures had low adherent ability and proliferation activity. Until now, our knowledge about how to prevent the differentiation of GCs into luteinized GCs in vitro culture has been limited. Thus, a culture system for GCs that inhibits the luteinization of GCs under culture conditions containing serum needs to be developed [11,12].

Establishing an in vitro culture system for non-luteinized GCs from sheep holds significant importance in scientific research. This study explores whether and how sheep GCs undergo luteinization under conventional in vitro culture conditions, with the goal of developing a culture system that preserves the physiological characteristics of GCs. Such a system will facilitate a deeper understanding of the regulatory mechanisms governing GCs and the development of early antral follicles during the estrous cycle.

## 2. Materials and Methods

### 2.1. Chemicals and Reagents

BAY-899 was obtained from MedChemExpres (Monmouth Junction, NJ, USA). Antibody specificity has been verified by peptides, and detailed results are shown in Supplementary Figure S1.

### 2.2. Isolation and Culture of GC

Healthy nonpregnant sheep (Han sheep, weighing 25–35 kg, *n* = 60) were slaughtered at an abattoir in a nearby city (Ulanqab, Inner Mongolia, China), and fresh ovaries were transported to the laboratory in saline supplemented with penicillin (10 U/mL) and streptomycin (10 µg/mL) at 35–37 °C within 2 h. GCs were collected from the follicular fluid of antral follicles (2–5 mm in diameter) and resuspended in Dulbecco’s modified Eagle medium/F12 medium (DMEM/F-12, HyClone, Shanghai, China) supplemented with 12% FBS (Clark, Richmond, VA, USA) and 1% penicillin/streptomycin (Biological Industries, Kibbutz Beit Haemek, Israel). Then, GCs were seeded in plates at the appropriate cell density for the study assays and incubated at 37 °C in a humidified 5% CO_2_ incubator.

### 2.3. Immunofluorescence Analysis

Immunofluorescence analysis was conducted to confirm the specificity of FSHR in GCs. Fresh GCs were seeded onto poly-L-lysine-coated slides at appropriate densities and cultured in DMEM/F12 media supplemented with 12% FBS for 48 h. The cells were then fixed with 4% paraformaldehyde, permeabilized with 0.1% Triton X-100 for 5 min, and blocked with 3% bovine serum albumin in PBS for 20 min. Subsequently, the cells were incubated overnight at 4 °C with the primary antibodies rabbit anti-FSHR (1:100, 22665-1-AP, Proteintech, Wuhan, China) and rabbit anti-STAR (1:100, bs-20388R, Bioss, Beijing, China). Detection was performed using Alexa Fluor^®^ 555 goat anti-rabbit secondary antibody (1:800, ab150078 Abcam, Cambridge, MA, USA) at 37° C for 1 h. Finally, nuclei were counterstained with DAPI (2 µg/mL, Abcam, Cambridge, MA, USA).

### 2.4. Western Blot

Total proteins were extracted from GCs using radioimmunoprecipitation assay buffer (CWBIO, Taizhou, China) supplemented with 1% protease inhibitor (CWBIO). The cells were kept on ice for 30 min to ensure protein integrity. Protein concentrations were determined using a BCA protein analysis kit (Thermo Fisher Scientific, Waltham, MA, USA) and subsequently diluted to a consistent concentration. Proteins were separated on a 12% sodium dodecyl sulfate polyacrylamide gel and transferred onto a 0.45 µm PVDF membrane (Merck Millipore, Darmstadt, Germany ). The membranes were blocked with blocking buffer (LI-COR Biosciences, Lincoln, NE, USA) for 1 h at room temperature to prevent nonspecific binding. They were then incubated overnight at 4 °C with primary antibodies, including mouse anti-α-tubulin antibody (1:10,000, T5168, Sigma, St. Louis, MO, USA), rabbit anti-FSHR (1:500, 22665-1-AP, Proteintech, Wuhan, China), and rabbit anti-STAR (1:500, bs-20388R, Bioss, Beijing, China). The membranes were subsequently incubated for 1 h at room temperature with secondary antibodies, specifically donkey anti-rabbit (1:10,000, 926-32213 LI-COR Biosciences, Lincoln, NE, USA) and donkey anti-mouse (1:10,000, 926-32212, LI-COR Biosciences, Lincoln, NE, USA). Visualization of the protein bands was performed using the Odyssey Infrared Imaging System (LI-COR Biosciences, Lincoln, NE, USA). α-Tubulin was used as the internal control. Western blot original images can be found in Supplementary File S1. 

### 2.5. Enzyme-Linked Immunosorbent Assay

The P4 concentration in the cell culture media was measured using an ELISA kit (E-OSEL-S0005, Elabscience, Wuhan, China). GCs were seeded at a density of 1 × 10^5^ viable cells per 2 mL of media per well in 6-well plates. Supernatants were collected at different time points (0, 12, 24, and 48 h) to assess P4 secretion. The sensitivity of the P4 assay was 0.01 ng/mL. The LH concentration in DMEM/F12 media, with and without FBS, was also determined using an ELISA kit (ESH0034, Wuhan Fine Biotech, Wuhan, China). The sensitivity of the LH assay was 0.9938 mIU/mL. Hormone concentrations were calculated using a standard curve, which was established from optical density values measured at 450 nm.

### 2.6. RT-qPCR

In the present study, RT-qPCR experiments were performed in accordance with the MIQE (Minimum Information for Publication of Quantitative Real-Time PCR Experiments) guidelines [13]. Briefly, after treatment, the cell culture media was discarded, and the cells were washed twice with PBS. Total RNA was extracted from cells using an AxyPrep total RNA preparation kit (Axygen, Union City, CA, USA) according to the manufacturer instructions. Then 500 µL lysis buffer was added to each well. The concentration, 260/230 ratio, and 260/280 ratio of the extracted total RNA were assessed using a Biotek Synergy HT microplate reader (BioTek, Charlotte, VT, USA), with ratios within the range of 2.0–2.2 were deemed acceptable for subsequent analysis. A total of 1 µg of RNA was mixed with 3 µL of Master Mix, which includes 5 × gDNA Eraser Buffer and gDNA Eraser to remove the genome DNA using gDNA Eraser (RR820A, TaKaRa, Dalian, China). The mixture was reverse transcribed to cDNA for gene expression analysis using the PrimeScriptTM RT reagent Kit (RR047A, TaKaRa, Dalian, China) containing PrimeScript RT Enzyme Mix I, RT Primer Mix, 5 × PrimeScript Buffer 2, and RNase Free dH2O. The reverse transcription was performed according to the manufacturer’s instructions: 15 min at 37 °C and 5 s at 85 °C for RT inactivation. TB Green^®^ Premix Ex TaqTM II (RR820A, TaKaRa, Dalian, China) was used in q-PCR assay to detect the gene expression. The amplification conditions were as follows: initial denaturation for 30 s at 95 °C and 40 cycles of 5 s at 95 °C and annealing for 34 s at 60 °C. A melt curve analysis was then performed at 60 °C. Detailed primer information was listed in Table 1. Non-template controls for Reverse Transcription and qPCR have been included in all reactions. The PCR products have been validated. The relative expression level of target genes was normalized to the expression level of β-actin for gene expression analysis using 2^^(–ΔΔCt)^ method. Data were presented as fold changes relative to the negative control group.

### 2.7. Cell Growth Curve

GCs were counted using a cell counter following various treatments. Each well of a 24-well plate was seeded with 10,000 GCs. After the cells adhered to the plate, their numbers were monitored and assessed using the cell counter over an 8-day period.

### 2.8. Statistical Analysis

Each experiment was performed in triplicate and the results are presented as the mean ± SEM. Data analysis and visualization were conducted using GraphPad Prism 5.0 (GraphPad, San Diego, CA, USA). One-way analysis of variance followed by Tukey’s post hoc test and independent sample *t*-tests were used to analyze the data. Statistical significance was determined at *p* < 0.05.

## 3. Results

### 3.1. Detection of FSHR and STAR Expression in Sheep GCs During Primary Culture

**Figure 1:**
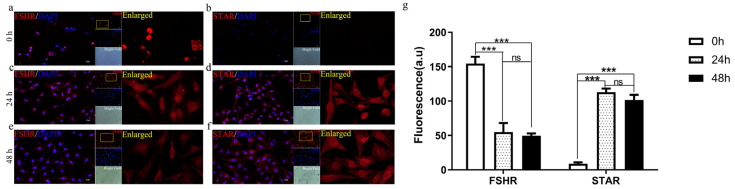
Localization of FSHR and STAR in sheep granulosa cells during primary culture. Intense immunostaining of FSHR (red) and STAR (red) proteins in sheep fresh GCs (**a**,**b**), and in primary GCs cultured in serum-containing media for 24 h (**c**,**d**) and 48 h (**e**,**f**). Cell nuclei were counterstained with DAPI (blue). The localization of FSHR and STAR in GCs is shown in the enlarged images marked by yellow boxs. Fluorescence intensity was quantified using ImageJ 1.54g (**g**) (*n* = 3). Bar = 10 μm. Values are expressed as mean ± SEM, *** *p* < 0.001, “ns” is the abbreviation of “no significance,” indicating no statistical significance in the difference between the two groups (*p* > 0.05).

This study employed cell immunofluorescence to examine the expression of FSHR and STAR proteins in fresh GCs (0 h), as well as in sheep GCs cultured in vitro for 24 and 48 h. The results showed that fresh GCs expressed FSHR, while STAR protein was absent (Figure 1a,b). After 24 and 48 h of in vitro culture, GCs continued to express FSHR, while STAR protein was detected (Figure 1c–e). Semi-quantitative analysis revealed that after 24 and 48 h of culture, FSHR protein significantly decreased (*p* < 0.001), whereas STAR protein significantly increased (*p* < 0.001) (Figure 1g).

### 3.2. Detection of Luteinization-Related Indicators in GCs During Primary Culture

**Figure 2:**
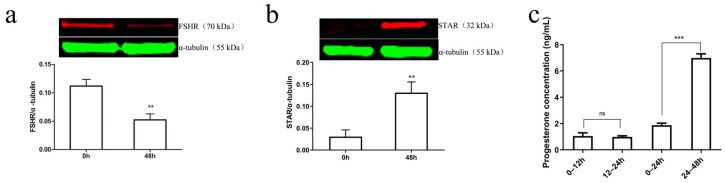
Detection of luteinization-related indicators in granulosa cells during primary culture. The protein levels of FSHR (**a**) and STAR (**b**) in primary GCs cultured in serum-containing media for 48 h were analyzed by immunoblotting and quantified. α-tubulin was used as an internal control. P4 (**c**) accumulation in the serum-containing media of primary GCs cultured in vitro for the same duration was measured. Values are expressed as mean ± SEM, ** *p* < 0.01, *** *p* < 0.001, “ns” is the abbreviation of “no significance,” indicating no statistical significance in the difference between the two groups (*p* > 0.05).

Protein immunoblotting and ELISA techniques were used to measure changes in the expression of luteinization-related molecules in primary GCs cultured in vitro for 48 h. The results showed that, compared to 0 h, the expression of the GC-specific receptor FSHR protein significantly decreased after 48 h of culture (*p* < 0.01) (Figure 2a), while STAR protein significantly increased (*p* < 0.01) (Figure 2b). No significant difference in P4 levels was observed between 12–24 h and 0–12 h, but P4 levels significantly increased between 24–48 h compared to 0–24 h (*p* < 0.001) (Figure 2c).

### 3.3. Inducing Factors of GC Luteinization During Primary Culture

**Figure 3:**
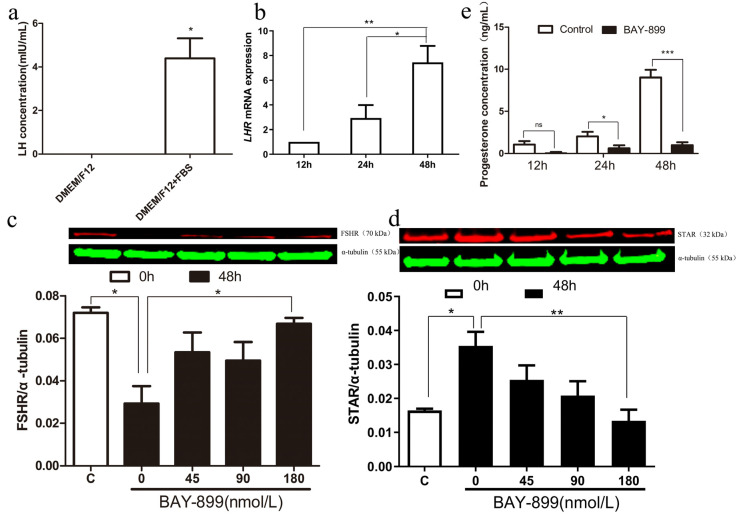
A preliminary study on the causes of luteinization and the role of BAY-899 in primary cultured granulosa cells. The LH (**a**) levels in serum-free and serum-containing media were measured by ELISA. The relative mRNA levels of LHR (**b**) in serum-containing media were measured at 12, 24, and 48 h. Primary sheep GCs were cultured in serum-containing media with or without BAY-899 (0–180 nmol/L) for 48 h. The cells were collected to assess FSHR (**c**) and STAR (**d**) protein expression by Western blotting. The P4 concentration (**e**) in the culture supernatants was determined by ELISA after treatment with or without 180 nmol/L BAY-899 for 12, 24, and 48 h. Values are presented as mean ± SEM, * *p* < 0.05, ** *p* < 0.01, *** *p* < 0.001.

To verify the presence of LH in serum, ELISA technology was used to measure LH levels in both serum-free and serum-containing media. The results showed that the serum-free media lacked LH, while the serum-containing media contained 4.39 mIU/mL of LH (*p* < 0.05) (Figure 3a). RT-qPCR was employed to assess changes in LHR mRNA expression in GCs during in vitro culture under serum-containing media conditions at 12, 24, and 48 h. The results indicated that LHR transcription levels gradually increased over time (*p* < 0.05) (Figure 3b), suggesting the involvement of the LH-LHR system in the spontaneous luteinization of GCs. Primary GCs were treated with different concentrations of the LHR antagonist BAY-899 (0, 45, 90, and 180 nmol/L) to examine changes in FSHR and STAR protein expression. The results showed that FSHR protein levels increased with higher concentrations of BAY-899, with the strongest effect observed at 180 nmol/L (*p* < 0.05) (Figure 3c). In contrast, STAR protein levels decreased in a concentration-dependent manner, with significant differences at 180 nmol/L BAY-899 (*p* < 0.01) (Figure 3d). Based on these findings, a concentration of 180 nmol/L was selected for further experiments on sheep GCs. ELISA was used to assess P4 secretion by GCs over 48 h in the presence of 180 nmol/L BAY-899. The results showed that, compared to the control group, BAY-899 significantly inhibited P4 secretion after 24 h (*p* < 0.05) and 48 h (*p* < 0.001) of in vitro culture (Figure 3e).

### 3.4. Effects of BAY-899 on Luteinization of Second-Generation GCs

**Figure 4:**
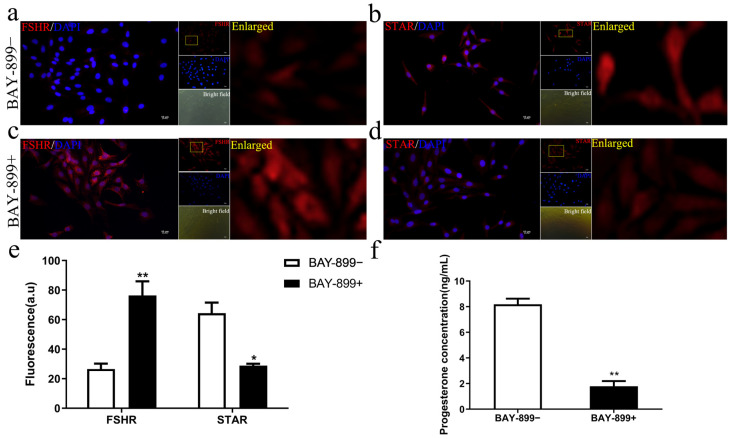
Effect of BAY-899 on the luteinization process of second-generation granulosa cells (GCs). Second-generation sheep GCs were incubated in serum-containing media with (BAY-899+) or without BAY-899 (BAY-899−) (180 nmol/L) for 48 h. Immunostaining of FSHR protein (red, (**a**,**c**)) and STAR (red, (**b**,**d**)) in second-generation GCs cultured in serum-containing media with (BAY-899+) or without BAY-899 (BAY-899−). Cell nuclei were counterstained with DAPI (blue). FSHR and STAR in second-generation GCs are highlighted by the yellow squares in enlarged images. Fluorescence intensity was measured using Image J software (**e**) (*n* = 3). Bar = 10 μm. Values are expressed as mean ± SEM, * *p* < 0.05, ** *p* < 0.01. The concentration of P4 (**f**) in the culture supernatants was measured by ELISA after second-generation GCs were treated with (BAY-899+) or without 180 nmol/L BAY-899 (BAY-899−) for 48 h. Values are mean ± SEM, ** *p* < 0.01.

To verify whether LHR antagonists can prevent GCs from undergoing luteinization, BAY-899 was added to the in vitro culture of GCs. By passaging, we compared the localization and expression of FSHR and STAR proteins in second-generation GCs, with or without BAY-899. The results showed that in the (BAY-899+) group, FSHR protein fluorescence was stronger compared to the (BAY-899−) group (Figure 4a,c), while STAR protein fluorescence was weaker (Figure 4b,d). Semi-quantitative analysis revealed that FSHR protein levels significantly increased in the (BAY-899+) group compared to the (BAY-899−) group (*p* < 0.01), while STAR protein levels were significantly decreased (*p* < 0.05) (Figure 4e). After 48 h in vitro culture, we measured and compared P4 levels in the culture media of both the (BAY-899+) and (BAY-899−) groups. The results showed that BAY-899 significantly inhibited P4 secretion compared to the (BAY-899−) group (*p* < 0.01) (Figure 4f). The same set of results, compared to the (BAY-899+) group, the (BAY-899−) group exhibits a significant reduction in FSHR expression and P4 production, accompanied by a marked increase in STAR expression.

### 3.5. Effects of BAY-899 on In Vitro Proliferation Activity of Fifth-Generation GCs

**Figure 5:**
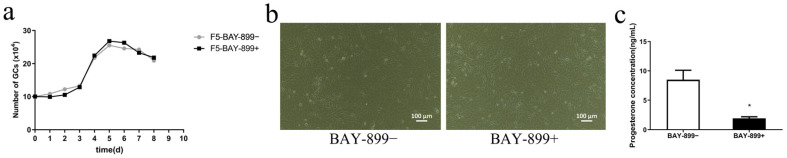
Effect of BAY-899 on the proliferative activity of fifth-generation granulosa cells (GCs) in vitro. Cell growth curves of fifth-generation GCs were determined using a cell counter after treatment with (BAY-899+) or without 180 nmol/L BAY-899 (BAY-899−) for 8 days (**a**). Morphological images of fifth-generation GCs cultured in vitro with (BAY-899+) or without BAY-899 (BAY-899−) are shown (**b**). The concentration of P4 (**c**) in the culture supernatants was measured by ELISA after fifth-generation GCs were treated with (BAY-899+) or without 180 nmol/L BAY-899 (BAY-899−) for 48 h. Values are mean ± SEM, * *p* < 0.05.

BAY-899 was added to in vitro cultured GCs up to the fifth generation to evaluate its effect on cell proliferation activity. The results showed that the cell growth curves of both the (BAY-899+) and (BAY-899−) groups were characteristic “S” shapes, indicating that the addition of exogenous BAY-899 did not affect the in vitro proliferation activity of sheep GCs (Figure 5a). Sheep ovarian GCs cultured with BAY-899 (BAY-899+) were stably passed to the fifth generation, and the adherent cells exhibited a typical cobblestone or spindle shape, as shown in Figure 5b. As shown in Figure 5c, BAY-899 treatment (BAY-899+) significantly suppresses P4 production in fifth-generation GCs (*p* < 0.01).

## 4. Discussion

GCs are the primary cell type used to investigate the processes involved in ovarian steroid hormone production and follicular development. To study the regulatory mechanisms underlying early antral follicle development, it is essential to use highly pure and viable GCs. GC differentiation within pre-ovulatory follicles is initiated by the LH surge before ovulation in vivo environment. Therefore, it is highly significant to investigate GC luteinization and establish a culture system with the capacity of maintaining the characteristics of GCs in vitro.

Currently, researchers have employed diverse methods to identify the luteinization of GCs cultured in vitro. McRae et al. were the first to suggest that an increased ability of GCs to secrete P4 and a decreased ability to secrete estrogen could serve as markers of luteinization. They identified STAR, CYP11A1, and 3β-HSD as key genes involved in luteinization [14]. Sugino et al. [15] further demonstrated that the expression of STAR in GCs significantly increased, while the expression of CYP19A1 significantly decreased, indicating that GCs undergo luteinization. This study examines the functional differences between ovine ovarian GCs and luteal cells. Through immunofluorescence staining and Western blot analysis, it identifies the expression of FSHR and STAR proteins in both fresh ovine GCs and those cultured in a serum-containing media, to determine whether differentiation has occurred. Additionally, the degree of luteinization was assessed by measuring P4 content in the culture media. The results showed that, compared to fresh GCs, FSHR protein expression significantly decreased, while STAR protein expression significantly increased after 48 h of culture in a serum-containing media. Furthermore, P4 content significantly increased within 48 h of in vitro culture, which aligns with the findings of Maoduo et al. [16]. FSHR is a specific marker of GCs, and its expression peaks during the small antral follicle stage before decreasing as the follicle matures [17]. Our findings suggest that fresh ovine GCs exhibit a marked decrease in FSHR, a characteristic protein of GCs, and a significant increase in STAR protein and P4 content after 48 h of culture in a conventional system containing serum, indicating that ovine GCs cultured in vitro undergo luteinization.

Scholars have explored various methods to block the luteinization of GCs in vitro. Some studies have used serum-free media to inhibit the luteinization of bovine GCs [18,19], but the formulation of serum-free media is complex and often contains androgen mimetics, which may interfere with studies on the effects of androgens on follicular development. Other research has utilized a tetracycline-inducible (Tet-on) 3G system to establish a GC line with a reversible proliferative state to prevent luteinization [20], although many immortalized cell lines lack steroidogenic activity [21]. We hypothesize that the spontaneous luteinization of GCs during in vitro culture may be due to the presence of exogenous LH or other LH-like substances in FBS, which can act as ligands for LHR and induce luteinization [22]. It is well known that FBS is obtained from pregnant cows in late gestation through cardiac puncture [23]. Human fetal serum collected at 20–22 weeks of gestation contains high levels of LH [24], suggesting that mammalian fetal serum may also contain detectable levels of LH. In this study, we observed that the LH content in media supplemented with serum was significantly higher compared to the basic culture media. GCs from both immature and mature human follicles exhibit low but measurable LHR expression. Interestingly, cumulus cells appear to upregulate LHR expression during the ovulation process [25]. Our study also found that after 48 h of in vitro culture, LHR mRNA expression in sheep GCs gradually increased. This finding aligns with previous in vitro studies on bovine GCs, where LHR expression increased at 48 h and remained elevated until 96 h. Moreover, the number of LHR-positive cells was significantly correlated with P4 secretion [22]. In comparison, our new system using the LHR antagonist BAY-899 exhibits distinct advantages. While serum-free conditions may alter basal cell physiology beyond luteinization inhibition, and the 3G system relies on genetic manipulation with potential off-target effects, BAY-899 specifically targets the LH receptor, a key mediator of luteinization signaling. This specificity makes it a more precise tool for studying luteinization mechanisms, complementing existing methods while addressing their limitations. Therefore, subsequent experiments will focus on further exploring the possibility of inhibiting GC differentiation through the LH-LHR signaling pathway.

In the present study, we observed that during the in vitro culture process, LH in serum activates the downstream signaling pathways that promote the luteinization of GCs by binding to and activating the LHR on GCs. The small molecule compound BAY-899, known for its inhibitory activity against LHRs in humans, rats, and crab-eating macaques, binds to a presumed rhodopsin-like binding pocket within the seven transmembrane regions of LHCGR through an allosteric mechanism [26]. In this study, various concentrations of BAY-899 were applied to primary early antral GCs from sheep. Notably, BAY-899 (180 nmol/L) significantly enhanced FSHR protein expression while reducing STAR protein expression. The in vitro characterization of BAY-899 revealed an IC50 for human LH of 185 nmol/L, which aligns with the results obtained in this study. Furthermore, at a concentration of 180 nmol/L, BAY-899 inhibited P4 secretion without affecting cell viability, maintaining P4 levels at a very low level. These results suggest that BAY-899 effectively inhibits the spontaneous luteinization of primary sheep ovarian GCs in vitro. Additionally, BAY-899 exhibited the same inhibitory effect on the luteinization of passaged GCs. The second-generation GCs in the maintained BAY-899 failed to differentiate, demonstrating that sustained BAY-899 exposure effectively maintains suppression of GC differentiation, when viewed from the perspective of the withdrawal group, shows that removal of BAY-899 permits the recovery of differentiation capacity. These findings confirm that continuous BAY-899 exposure inhibits GC differentiation, while removing BAY-899 clearly restores this differentiation capacity.

LHR, a G protein-coupled receptor located on GCs, plays a central role in regulating P4 production [27]. The activation of LHR triggers signaling pathways such as the cyclic adenosine monophosphate (cAMP) pathway [28], which in turn activates steroidogenic enzymes, including STAR [29], to facilitate sex hormone production. BAY-899, acting as an LHR antagonist, reduces LHR activity, leading to the downregulation of the cAMP pathway. Given that cAMP is a key regulatory factor in steroidogenesis, its reduction directly inhibits the expression and activity of STAR protein, thereby impacting steroid biosynthesis. STAR’s main function is to facilitate the transport of cholesterol from the cytoplasm to the mitochondria, a crucial step in P4 synthesis. Therefore, we hypothesize that BAY-899 may indirectly inhibit P4 synthesis by decreasing STAR protein expression. In conclusion, this study successfully established an in vitro culture system capable of maintaining the characteristics of sheep ovarian GCs. This system utilizes a culture media containing LHR antagonists to prevent the natural transition of GCs into luteinization, allowing for normal growth, development, and proliferation of cells in vitro.

## 5. Conclusions

It is demonstrated that sheep GCs undergo spontaneous luteinization induced by LH-LHR signaling in serum-containing media systems. As far as we know, it is the first to establish a culture system that preserves the characteristics of GCs using the LHR antagonist BAY-899, providing an ideal cellular model for in-depth studies on the behavior and responses of sheep GCs under various physiological conditions, as well as the mechanisms underlying early antral follicle development.

## Figures and Tables

**Table 1:** Primer sequence information.

Primer	Sequence (5′→3′)	Length (bp)	Accession Number
β-actin	F: CCATCGGCAATGAGCGGT R: CGTGTTGGCTAGAGGTC	146	NM_001009784.3
LHR	F: TGCTTACCCAAGACACTCR: ATCAGCCAAATCAGGAC	101	NM_001278566.2

## Data Availability

The original contributions presented in the study are included in the article; further inquiries can be directed at the corresponding author.

## Supplementary Materials

The following supporting information can be downloaded at https://www.mdpi.com/article/10.3390/biom15091280/s1. Figure S1: Western blot image of isotype control antibody (IgG raised in rabbit). File S1: The original images for Western blots.
