# Supplementary material for: A Novel Culture System for Inhibiting In Vitro Differentiation of Ovine Granulosa Cells
Source: Biomolecules. 2025 Sep 4;15(9):1280. doi: 10.3390/biom15091280 (PMC12467111; doi:10.3390/biom15091280)
Supplement: Supplementary file 1 [file biomolecules-15-01280-s001.zip › Figure S1.pdf]

Supplementary Files

Figure S1. Validation of antibody specificity.

The supplementary isotype control experiments were performed following the same protocols as the original antibody-based assays. All samples were processed in parallel with the experimental groups under identical conditions to ensure consistency. The results of the supplementary experiments strongly support the specificity of our antibody-based detection methods. As shown in Figure S1, no detectable bands were observed at the expected molecular weight of the target protein in samples treated with isotype control antibodies, contrasting sharply with the distinct bands obtained using the specific primary antibody. These new findings effectively rule out potential non-specific binding, providing additional validation for the specificity of our antibody and strengthening the overall reliability of our results.

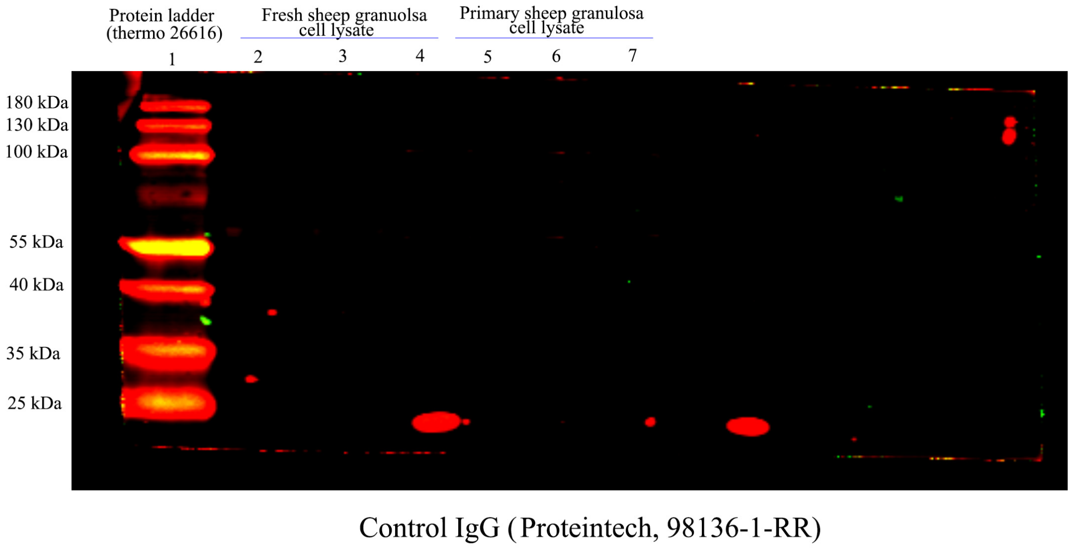

Figure S1. Validation of antibody specificity by isotype control in Western blot analysis. The isotype control antibody used in this experiment was sourced from Proteintech (catalog #98136-1-RR). It was a polyclonal antibody of the IgG isotype, matching the host species (rabbit) of the target primary antibody. Lane 1: Pre-stained protein marker (Thermo Fisher Scientific, Cat. No. 26616), with key molecular weights labeled (25, 35, 40, 55, 100, 130, and 180 kDa). Lanes 2-4: Fresh sheep granulosa cells lysate. Lanes 5-7: Primary sheep granulosa cells lysate.
