# Supplementary figures and images for: A Novel Culture System for Inhibiting In Vitro Differentiation of Ovine Granulosa Cells
Source: Biomolecules. 2025 Sep 4;15(9):1280. doi: 10.3390/biom15091280 (PMC12467111; doi:10.3390/biom15091280)

Figure 2a

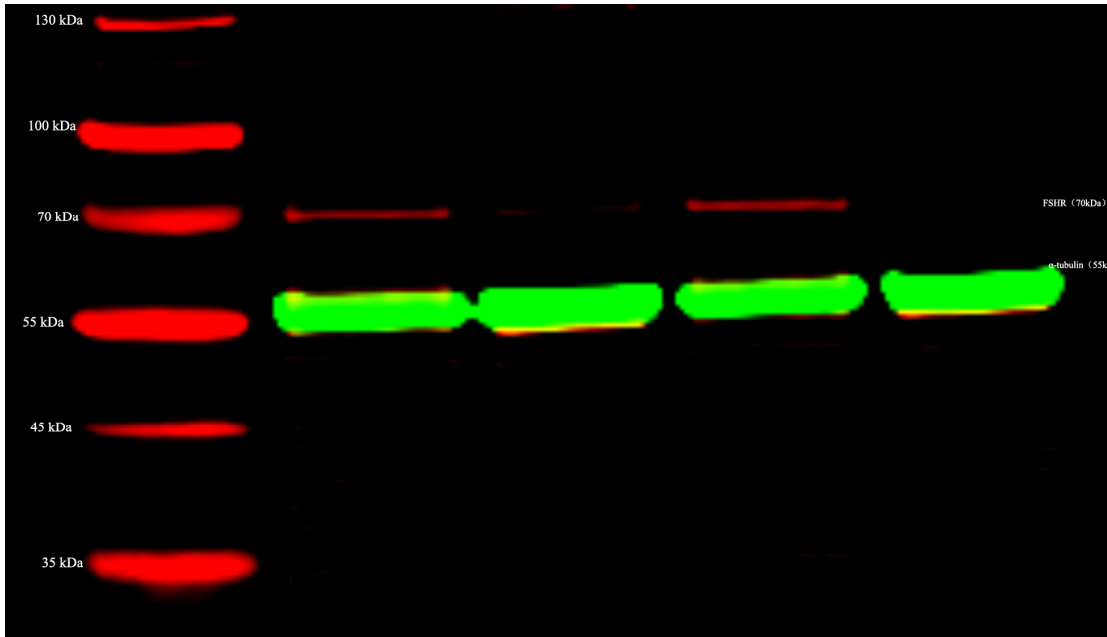

Figure 2b

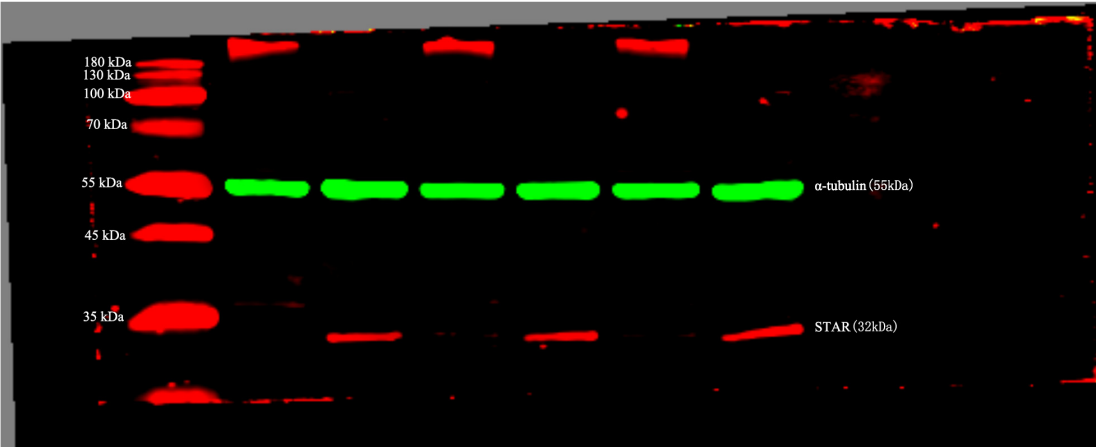

Figure 3c

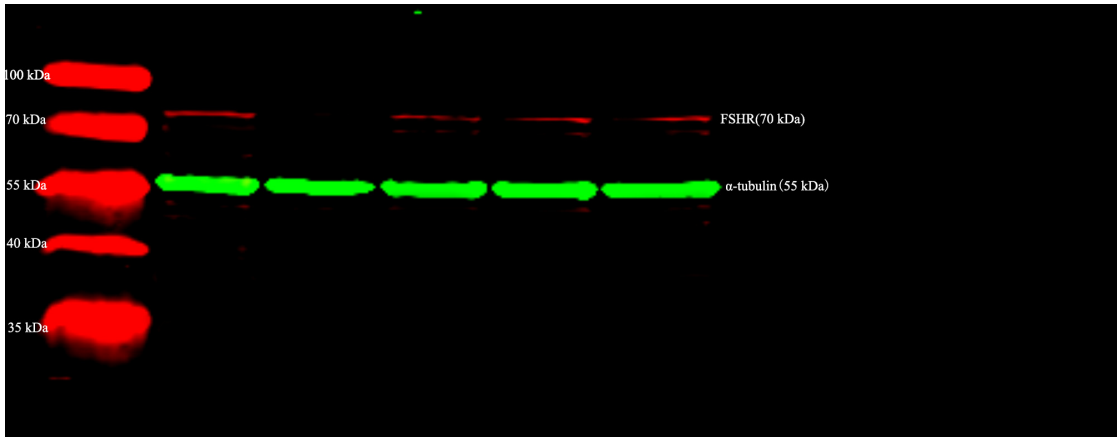

Figure 3d

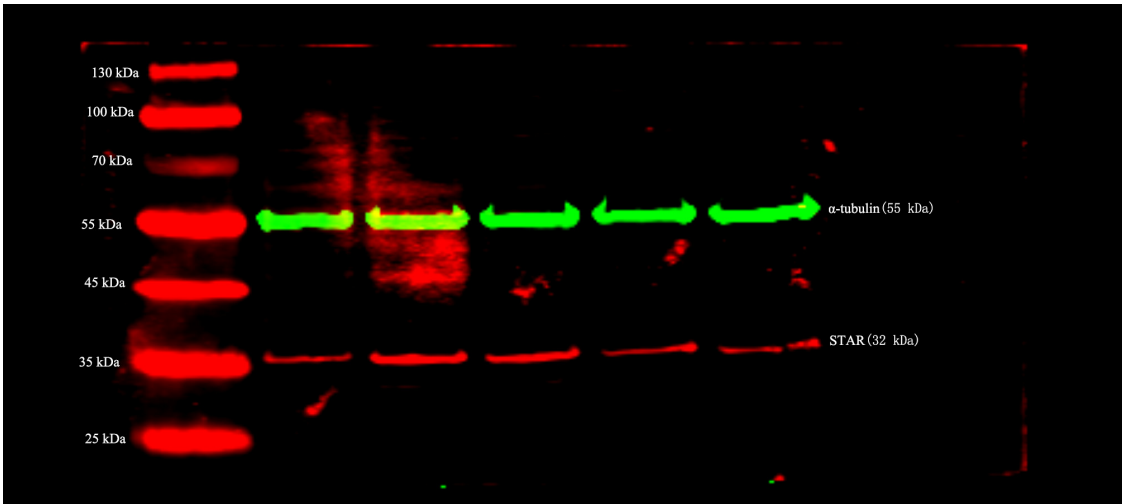

Supplement: Supplementary file 1 [file biomolecules-15-01280-s001.zip › File S1-Original Western Blots.pdf]
